# Chlorido(5-formyl-2-hydroxy­phenyl-κ*C* ^1^)mercury(II)

**DOI:** 10.1107/S1600536809043529

**Published:** 2009-10-28

**Authors:** Ze-Bao Zheng, Shi-Ying Ma, Ren-Tao Wu, Yin-Feng Han, Jing-Rong Lu

**Affiliations:** a Department of Chemistry and Environmental Science, Taishan University, 271021 Taian, Shandong, People’s Republic of China

## Abstract

In the planar (r.m.s. deviation = 0.027 Å) title compound, [Hg(C_7_H_5_O_2_)Cl], the Hg^II^ atom shows a typical linear coordination by a C atom of the benzene ring and a Cl atom. Inter­molecular O—H⋯O hydrogen bonds are present in the crystal structure, resulting in chains propagating along the *b* axis. The crystal studied was a non-merohedral twin, with a twin ratio of 0.802 (2):0.198 (2).

## Related literature

For general background to the use of cyclo­metallated compounds in synthesis, catalysis and materials, see: Gruter *et al.* (1995 ▶); Dupont *et al.* (2005 ▶). For related structures and the synthesis of related cyclo­mercurated compounds, see: Xu *et al.* (2009 ▶). For the preparation of cyclo­mercurated compounds, see: Ryabov *et al.* (2003 ▶); Wu *et al.* (2001 ▶).
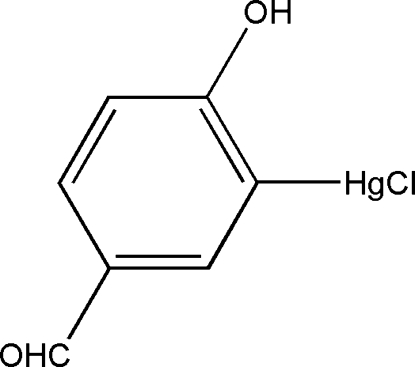


## Experimental

### 

#### Crystal data

[Hg(C_7_H_5_O_2_)Cl]
*M* *_r_* = 357.15
Monoclinic, 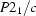

*a* = 4.1004 (10) Å
*b* = 14.842 (3) Å
*c* = 14.116 (3) Å
β = 106.657 (6)°
*V* = 823.0 (3) Å^3^
*Z* = 4
Mo *K*α radiation
μ = 18.97 mm^−1^
*T* = 295 K
0.20 × 0.18 × 0.16 mm

#### Data collection

Bruker SMART CCD diffractometer
Absorption correction: multi-scan (*SADABS*; Sheldrick, 1996 ▶) *T*_min_ = 0.116, *T*_max_ = 0.151
4116 measured reflections
1424 independent reflections
1333 reflections with *I* > 2σ(*I*)
*R*_int_ = 0.039

#### Refinement

*R*[*F* ^2^ > 2σ(*F* ^2^)] = 0.039
*wR*(*F* ^2^) = 0.103
*S* = 1.09
1424 reflections
101 parameters
H-atom parameters constrained
Δρ_max_ = 2.05 e Å^−3^
Δρ_min_ = −1.73 e Å^−3^

### 

Data collection: *SMART* (Siemens, 1996 ▶); cell refinement: *SAINT* (Siemens, 1996 ▶); data reduction: *SAINT*; program(s) used to solve structure: *SHELXS97* (Sheldrick, 2008 ▶); program(s) used to refine structure: *SHELXL97* (Sheldrick, 2008 ▶); molecular graphics: *SHELXTL* (Sheldrick, 2008 ▶); software used to prepare material for publication: *SHELXTL* and *PLATON* (Spek, 2009 ▶).

## Supplementary Material

Crystal structure: contains datablocks global, I. DOI: 10.1107/S1600536809043529/hb5153sup1.cif

Structure factors: contains datablocks I. DOI: 10.1107/S1600536809043529/hb5153Isup2.hkl

Additional supplementary materials:  crystallographic information; 3D view; checkCIF report

## Figures and Tables

**Table:** 

Hg1—C3	2.058 (13)
Hg1—Cl1	2.326 (4)

**Table:** 

C3—Hg1—Cl1	179.1 (4)

**Table 2:** Hydrogen-bond geometry (Å, °)

*D*—H⋯*A*	*D*—H	H⋯*A*	*D*⋯*A*	*D*—H⋯*A*
O2—H2⋯O1^i^	0.82	1.91	2.727 (16)	172

Symmetry code: (i) 
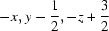
.

**Fig. 1.**
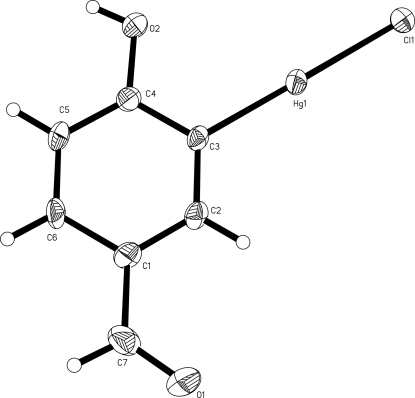
The molecular structure of (I), with displacement ellipsoids drawn at the 30% probability level.

**Fig. 2.**
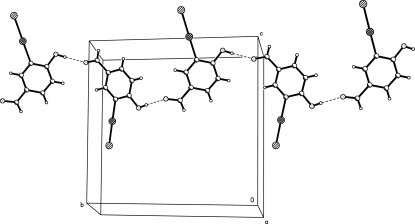
Partial view of the crystal packing showing the formation of the one-dimensional chain structure formed by the intermolecular O—H···O hydrogen bonds.

## supplementary crystallographic information

### Comment

Cyclometallated compounds have attracted much research interest owing to theirs utility in synthesis, catalysis and materials (Gruter *et al.*, 1995; Dupont *et al.*, 2005). Among them, cyclomercurated compounds are easy to prepare through a C–H activation process and are stable but reasonably reactive (Wu *et al.*, 2001; Ryabov *et al.*, 2003).

In the planar title compound (Fig. 1), the mercury(II) atom shows a typical linear coordination geometry with a carbon atom of the benzene ring and the chloride atom in *trans* position. O2–Hg1 distance (3.047 (2) Å) is much longer than those of the related Hg(II) complex (Xu *et al.*, 2009). The C–Hg and Hg–Cl bond distances are within normal ranges. The C3–Hg1–Cl1 angle is 179.1 (4) °. Intermolecular O—H···O hydrogen bonds are present in the crystal structure (Table 1), resulting in a one-dimensional supramolecular architecture (Fig.2).

### Experimental

The title compound was prepared from the *p*-hydroxybenzaldehyde with Hg(OAc)_2_ and subsequent treatment with LiCl and recrystallized from dichloromethane-petroleum ether solution at room temperature to give (I) as colorless crystals suitable for single-crystal X-ray diffraction.

### Refinement

All H atoms were placed in geometrically idealized positions, with C—H = 0.93–0.96 Å, O—H = 0.82–0.85 Å and N—H = 0.86 Å. *U*_iso_(H) = 1.2*U*_eq_(C,*N*), and 1.5*U*_eq_(O).

The structure is a non-merohdral twin. The twin law, as given by *PLATON* (Spek, 2009), is (-1 0 0, 0 - 1 0, 2 0 1), which lowered the R1 index from 0.116 to 0.039.

### Crystal data

**Table:**

[Hg(C_7_H_5_O_2_)Cl]	*F*(000) = 640
*M**_r_* = 357.15	*D*_x_ = 2.882 Mg m^−^^3^
Monoclinic, *P*2_1_/*c*	Mo *K*α radiation, λ = 0.71073 Å
Hall symbol: -P 2ybc	Cell parameters from 2640 reflections
*a* = 4.1004 (10) Å	θ = 2.7–29.5°
*b* = 14.842 (3) Å	µ = 18.97 mm^−^^1^
*c* = 14.116 (3) Å	*T* = 295 K
β = 106.657 (6)°	Block, colorless
*V* = 823.0 (3) Å^3^	0.20 × 0.18 × 0.16 mm
*Z* = 4	

### Data collection

**Table:**

Bruker SMART CCD diffractometer	1424 independent reflections
Radiation source: fine-focus sealed tube	1333 reflections with *I* > 2σ(*I*)
graphite	*R*_int_ = 0.039
φ and ω scans	θ_max_ = 25.1°, θ_min_ = 1.4°
Absorption correction: multi-scan (*SADABS*; Sheldrick, 1996)	*h* = −4→4
*T*_min_ = 0.116, *T*_max_ = 0.151	*k* = −12→17
4116 measured reflections	*l* = −16→15

### Refinement

**Table:**

Refinement on *F*^2^	Primary atom site location: structure-invariant direct methods
Least-squares matrix: full	Secondary atom site location: difference Fourier map
*R*[*F*^2^ > 2σ(*F*^2^)] = 0.039	Hydrogen site location: inferred from neighbouring sites
*wR*(*F*^2^) = 0.103	H-atom parameters constrained
*S* = 1.09	*w* = 1/[σ^2^(*F*_o_^2^) + (0.0429*P*)^2^ + 12.0653*P*] where *P* = (*F*_o_^2^ + 2*F*_c_^2^)/3
1424 reflections	(Δ/σ)_max_ = 0.001
101 parameters	Δρ_max_ = 2.05 e Å^−^^3^
0 restraints	Δρ_min_ = −1.73 e Å^−^^3^

### Special details

**Table:**

Geometry. All e.s.d.'s (except the e.s.d. in the dihedral angle between two l.s. planes) are estimated using the full covariance matrix. The cell e.s.d.'s are taken into account individually in the estimation of e.s.d.'s in distances, angles and torsion angles; correlations between e.s.d.'s in cell parameters are only used when they are defined by crystal symmetry. An approximate (isotropic) treatment of cell e.s.d.'s is used for estimating e.s.d.'s involving l.s. planes.
Refinement. Refinement of *F*^2^ against ALL reflections. The weighted *R*-factor *wR* and goodness of fit *S* are based on *F*^2^, conventional *R*-factors *R* are based on *F*, with *F* set to zero for negative *F*^2^. The threshold expression of *F*^2^ > σ(*F*^2^) is used only for calculating *R*-factors(gt) *etc*. and is not relevant to the choice of reflections for refinement. *R*-factors based on *F*^2^ are statistically about twice as large as those based on *F*, and *R*- factors based on ALL data will be even larger.

### Fractional atomic coordinates and isotropic or equivalent isotropic displacement parameters (Å^2^)

**Table:**

	*x*	*y*	*z*	*U*_iso_*/*U*_eq_	
Hg1	0.32843 (16)	0.87897 (3)	0.53385 (4)	0.0345 (2)	
Cl1	0.5374 (10)	0.9140 (2)	0.4016 (3)	0.0405 (8)	
O1	0.093 (5)	1.0269 (8)	0.8767 (9)	0.076 (4)	
O2	0.069 (4)	0.6993 (7)	0.5877 (8)	0.055 (3)	
H2	0.0023	0.6493	0.5982	0.083*	
C1	0.031 (5)	0.8833 (8)	0.8033 (11)	0.038 (3)	
C2	0.131 (4)	0.9074 (9)	0.7223 (10)	0.033 (3)	
H2A	0.1898	0.9671	0.7153	0.040*	
C3	0.148 (4)	0.8460 (9)	0.6511 (9)	0.029 (3)	
C4	0.052 (4)	0.7564 (10)	0.6613 (10)	0.036 (3)	
C5	−0.063 (5)	0.7317 (9)	0.7410 (11)	0.042 (3)	
H5	−0.1288	0.6724	0.7467	0.051*	
C6	−0.081 (4)	0.7932 (10)	0.8110 (10)	0.039 (4)	
H6	−0.1642	0.7766	0.8631	0.047*	
C7	−0.007 (6)	0.9495 (12)	0.8760 (11)	0.059 (5)	
H7	−0.1119	0.9319	0.9235	0.070*	

### Atomic displacement parameters (Å^2^)

**Table:**

	*U*^11^	*U*^22^	*U*^33^	*U*^12^	*U*^13^	*U*^23^
Hg1	0.0437 (3)	0.0258 (3)	0.0364 (3)	−0.0009 (2)	0.0153 (3)	0.0038 (2)
Cl1	0.050 (2)	0.0390 (18)	0.0357 (17)	−0.0049 (17)	0.0181 (16)	−0.0012 (15)
O1	0.133 (14)	0.035 (6)	0.067 (8)	−0.005 (8)	0.040 (9)	−0.009 (6)
O2	0.099 (11)	0.028 (5)	0.047 (6)	−0.014 (6)	0.033 (7)	−0.007 (5)
C1	0.050 (10)	0.022 (7)	0.043 (8)	0.011 (6)	0.015 (7)	0.002 (5)
C2	0.036 (8)	0.021 (6)	0.044 (8)	0.008 (6)	0.013 (6)	0.008 (6)
C3	0.032 (7)	0.021 (6)	0.030 (7)	0.002 (6)	0.003 (5)	0.007 (5)
C4	0.042 (8)	0.030 (7)	0.029 (7)	−0.003 (6)	0.002 (6)	0.003 (6)
C5	0.061 (10)	0.021 (6)	0.047 (8)	−0.005 (7)	0.020 (8)	0.008 (6)
C6	0.052 (9)	0.034 (8)	0.034 (7)	−0.005 (7)	0.014 (7)	0.016 (6)
C7	0.096 (15)	0.048 (10)	0.038 (9)	0.017 (10)	0.027 (10)	0.009 (7)

### Geometric parameters (Å, °)

**Table:**

Hg1—C3	2.058 (13)	C2—C3	1.37 (2)
Hg1—Cl1	2.326 (4)	C2—H2A	0.9300
O1—C7	1.22 (2)	C3—C4	1.405 (19)
O2—C4	1.357 (17)	C4—C5	1.39 (2)
O2—H2	0.8193	C5—C6	1.36 (2)
C1—C2	1.37 (2)	C5—H5	0.9300
C1—C6	1.428 (19)	C6—H6	0.9300
C1—C7	1.46 (2)	C7—H7	0.9300
			
C3—Hg1—Cl1	179.1 (4)	O2—C4—C3	115.9 (13)
C4—O2—H2	109.5	C5—C4—C3	120.1 (13)
C2—C1—C6	119.2 (13)	C6—C5—C4	120.8 (13)
C2—C1—C7	121.9 (13)	C6—C5—H5	119.6
C6—C1—C7	118.4 (15)	C4—C5—H5	119.6
C1—C2—C3	121.8 (13)	C5—C6—C1	119.2 (13)
C1—C2—H2A	119.1	C5—C6—H6	120.4
C3—C2—H2A	119.1	C1—C6—H6	120.4
C2—C3—C4	118.7 (13)	O1—C7—C1	122.3 (17)
C2—C3—Hg1	122.4 (10)	O1—C7—H7	118.8
C4—C3—Hg1	118.8 (10)	C1—C7—H7	118.8
O2—C4—C5	123.9 (13)		
			
C6—C1—C2—C3	−4(2)	O2—C4—C5—C6	−178.4 (16)
C7—C1—C2—C3	−176.1 (16)	C3—C4—C5—C6	−1(2)
C1—C2—C3—C4	1(2)	C4—C5—C6—C1	−2(3)
C1—C2—C3—Hg1	−175.8 (12)	C2—C1—C6—C5	4(2)
C2—C3—C4—O2	179.0 (14)	C7—C1—C6—C5	176.7 (16)
Hg1—C3—C4—O2	−3.9 (18)	C2—C1—C7—O1	−11 (3)
C2—C3—C4—C5	1(2)	C6—C1—C7—O1	177.2 (19)
Hg1—C3—C4—C5	178.5 (12)		

### Hydrogen-bond geometry (Å, °)

**Table:**

*D*—H···*A*	*D*—H	H···*A*	*D*···*A*	*D*—H···*A*
O2—H2···O1^i^	0.82	1.91	2.727 (16)	172

Symmetry codes: (i) −*x*, *y*−1/2, −*z*+3/2.
